# Functional gene-guided enrichment plus in situ microsphere cultivation enables isolation of new crucial ureolytic bacteria from the rumen of cattle

**DOI:** 10.1186/s40168-023-01510-4

**Published:** 2023-04-15

**Authors:** Sijia Liu, Zhongtang Yu, Huiyue Zhong, Nan Zheng, Sharon Huws, Jiaqi Wang, Shengguo Zhao

**Affiliations:** 1 State Key Laboratory of Animal Nutrition, Institute of Animal Sciences, Chinese Academy of Agricultural Sciences, No. 2 Yuanmingyuan West Road Haidian, Beijing,, 100193 China; 2 College of Pastoral Agriculture Science and Technology, Lanzhou University, Lanzhou, 730020 China; 3 Department of Animal Sciences, The Ohio State University, Columbus, OH 43210 USA; 4 School of Biological Sciences and Institute for Global Food Security, 19 Chlorine Gardens, Queen’s University Belfast, Belfast, UK

**Keywords:** Ureolytic bacteria, Urease, Agarose microsphere embedding, In situ cultivation, Isolation, Rumen

## Abstract

**Background:**

Ruminants can utilize urea as a dietary nitrogen source owing to their ability to recycle urea-N back to the rumen where numerous ureolytic bacteria hydrolyze urea into ammonia, which is used by numerous bacteria as their nitrogen source. Rumen ureolytic bacteria are the key microbes making ruminants the only type of animals independent of pre-formed amino acids for survival, thus having attracted much research interest. Sequencing-based studies have helped gain new insights into ruminal ureolytic bacterial diversity, but only a limited number of ureolytic bacteria have been isolated into pure cultures or studied, hindering the understanding of ureolytic bacteria with respect to their metabolism, physiology, and ecology, all of which are required to effectively improve urea-N utilization efficiency.

**Results:**

We established and used an integrated approach, which include urease gene (*ure*C) guided enrichment plus in situ agarose microsphere embedding and cultivation under rumen-simulating conditions, to isolate ureolytic bacteria from the rumen microbiome. We optimized the dilutions of the rumen microbiome during the enrichment, single-cell embedding, and then in situ cultivation of microsphere-embedded bacteria using dialysis bags placed in rumen fluid. Metabonomic analysis revealed that the dialysis bags had a fermentation profile very similar to the simulated rumen fermentation. In total, we isolated 404 unique strains of bacteria, of which 52 strains were selected for genomic sequencing. Genomic analyses revealed that 28 strains, which were classified into 12 species, contained urease genes. All these ureolytic bacteria represent new species ever identified in the rumen and represented the most abundant ureolytic species. Compared to all the previously isolated ruminal ureolytic species combined, the newly isolated ureolytic bacteria increased the number of genotypically and phenotypically characterized ureolytic species by 34.38% and 45.83%, respectively. These isolated strains have unique genes compared to the known ureolytic strains of the same species indicating their new metabolic functions, especially in energy and nitrogen metabolism. All the ureolytic species were ubiquitous in the rumen of six different species of ruminants and were correlated to dietary urea metabolism in the rumen and milk protein production. We discovered five different organizations of urease gene clusters among the new isolates, and they had varied approaches to hydrolyze urea. The key amino acid residues of the UreC protein that potentially plays critical regulatory roles in urease activation were also identified.

**Conclusions:**

We established an integrated methodology for the efficient isolation of ureolytic bacteria, which expanded the biological resource of crucial ureolytic bacteria from the rumen. These isolates play a vital role in the incorporation of dietary nitrogen into bacterial biomass and hence contribute to ruminant growth and productivity. Moreover, this methodology can enable efficient isolation and cultivation of other bacteria of interest in the environment and help bridge the knowledge gap between genotypes and phenotypes of uncultured bacteria.

Video abstract

**Supplementary Information:**

The online version contains supplementary material available at 10.1186/s40168-023-01510-4.

## Background

In most mammalian species, a large amount of endogenous urea produced in the liver is excreted via urine. As a unique group of animals with respect to N utilization, ruminants allow for constant recycling of urea back to the gastrointestinal tract, particularly the rumen, where urea-N can be used in de novo synthesis of amino acids and then microbial protein, which serves as the main N source (up to 80%) for ruminants [1]. In ruminants, 40–80% of the urea produced in the liver return to the gastrointestinal tract, especially the rumen [2]. Urea is hydrolyzed to carbon dioxide and ammonia by urease produced by ureolytic bacteria, and much of the ammonia is used as an N source by numerous rumen bacteria for microbial protein synthesis (MPS) [3]. Microbial protein is the major metabolizable N for ruminant milk and meat production. Taking advantage of this unique ability, many ruminant livestock producers use urea to partially replace dietary protein to reduce feeding costs [4]. Urease (EC 3.5.1.5) produced by rumen ureolytic bacteria is responsible for the urea hydrolysis in the rumen. Because urea hydrolysis mediated by urease is rather rapid, ammonia re-absorption through the rumen wall increases, which increases urine urea excretion and leads to poor efficiency of urea-N utilization in ruminants [5, 6] and environmental pollution [7]. Therefore, ureolytic bacteria in the rumen have attracted much research interest [8, 9].

Both cultivation-based and cultivation-independent studies have attempted to reveal the diversity and functions of rumen ureolytic bacteria. Most of the cultured rumen ureolytic bacteria were obtained in the last century by traditional platting cultivation, which is laborious and time-consuming. Only several species of rumen ureolytic bacteria have been isolated in previous studies [10–19] or some of them have not been comprehensively characterized and published, but high-throughput sequencing technologies have revealed a high diversity of ureolytic bacteria [5], nearly 600 operational taxonomic units, which are defined at 97% DNA sequence similarity of the *ure*C gene (the alpha subunit of urease gene), a genetic marker of urease [20–22]. Although such sequencing-based studies helped gain some new insights into the diversity and distributions of ureolytic bacteria in the rumen ecosystem [5, 23], their metabolism, physiology, and ecology remain poorly understood. Isolation and characterization of rumen ureolytic bacteria are critical to directly assessing and definitively defining their essential biological processes and features, which are required to inform new, efficient, feasible interventions to improve urea utilization efficiency.

It is difficult to isolate new ureolytic bacteria from the rumen because they are strictly anaerobic and present at relatively low abundance. Additionally, we know little about their requirement for nutrients and growth factors to formulate the appropriate media and cultivation conditions. Moreover, many microbes require cross-feeding or close interactions with other community members for growth [24, 25]. In situ cultivation simulates the rumen conditions for bacteria and overcomes the drawback of plating, facilitating, or enabling isolation of bacteria as individual isolates. Serial dilution of source samples can help isolate predominant bacteria [26]. Researchers have developed several novel techniques, such as ichip [27], culturomics [28], single-cell isolation [29], and microfluidic droplets [30], to increase the success to isolate bacteria that are difficult to culture from different environments. However, these techniques require expensive equipment, and the isolation is not targeted for a specific functional group. Additionally, due to their low abundance in the rumen [5, 10], enrichment of ureolytic bacteria prior to isolation would help subsequent isolation, but they cannot be effectively enriched using a selective carbon or energy source. In this study, we integrated *ure*C gene-guided enrichment, embedding single cells in agarose microspheres, and in situ cultivation to isolate ureolytic bacteria from the rumen of dairy cows. We then sequenced the genomes of the isolated ureolytic bacteria to determine their diversity, distribution, and identified urease gene clusters and activities.

## Results

### An integrated method facilitates targeting isolation of ureolytic bacteria

**Fig. 1:**
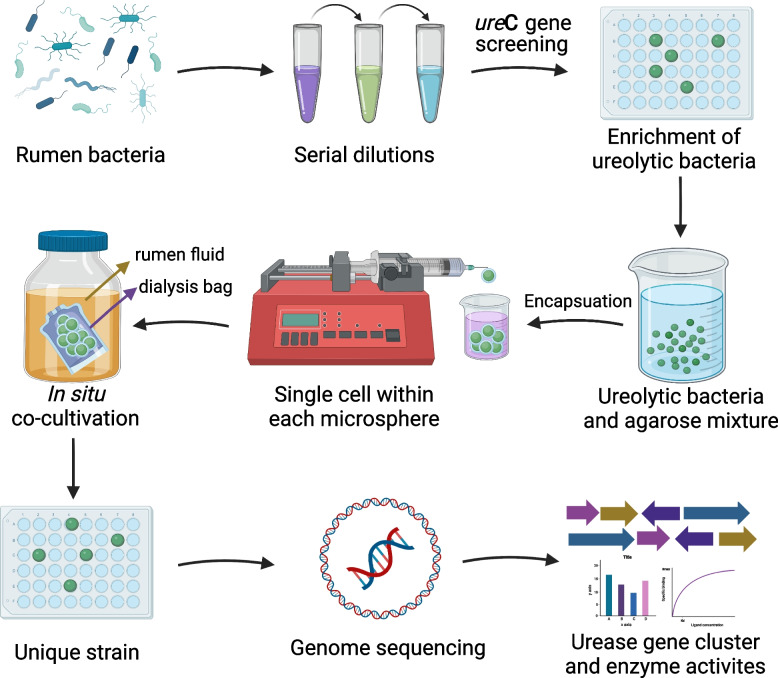
Workflow of the enrichment, isolation, and genomic characterization of anaerobic ureolytic bacteria from the rumen. Firstly, rumen microbiota samples were serially diluted and distributed into 96-well plates. After incubation, the wells with growth of ureolytic bacteria were identified by urease gene (*ure*C) specific PCR. Secondly, enriched ureolytic bacterial cells were serially diluted and embedded within agarose microspheres (aimed for one cell per microsphere). Thirdly, agarose microspheres were placed into dialysis bags that were placed into a rumen-simulating system for incubation. Finally, single strains in each microsphere were identified by 16S rRNA gene sequencing and subjected to whole-genome sequencing and analysis

We established an isolation method to help isolate rumen ureolytic bacteria by combining *ure*C-guided enrichment, embedding single cells in agarose microspheres, cultivation under rumen-simulating conditions, and genomic characterization (Fig. 1, detailed in Methods). This integrated method had several advantages. The use of 96-well plates increased throughput, *ure*C-specific PCR helped select enriched ureolytic bacteria, embedding in agarose microspheres facilitated simulated in situ incubation to allow those bacteria that would require interaction with other bacteria to grow, and genomic sequencing supported characterization of the urease gene clusters. We optimized the dilution of the inoculum (rumen fluid) and then the enriched ureolytic bacterial cultures to maximize the embedding of single cells into agarose microspheres (Supplementary Fig. 1). After incubation of the serially diluted rumen fluid for 24 h, *ure*C-specific PCR test showed that only some wells contained ureolytic bacteria beyond the 10^–1^ dilution, and no ureolytic bacteria could be detected at 10^–5^ or higher dilution (Supplementary Fig. 1 A). For the rumen fluid samples used in the present study, 10^–4^ dilution resulted in the most probable isolation of ureolytic bacteria into single strains and thus selected as the optimal dilution for the enriched ureolytic bacteria.

We diluted the enriched ureolytic bacteria, embedded them into agarose microspheres, and then incubated them in a rumen-simulating in situ system that allowed cross-feeding between ureolytic bacteria embedded in agarose microspheres inside dialysis bags and rumen microbiota outside the dialysis bags. The average diameter (± SD) of the agarose microspheres was 3.4 ± 0.1 mm (Supplementary Fig. 1 B). Amplification and sequencing analyses of the 16S rRNA genes of the cultures in individual agarose microspheres showed that with increasing dilution, the percentage of agarose microspheres that had bacteria decreased (Supplementary Fig. 1 C), while that of agarose microspheres that had single cells increased (Supplementary Fig. 1 D). Embedding of 100% single cells resulted from 10^–9^ and higher dilutions (Supplementary Fig. 1 D). To maximize the isolation of single strains from individual agarose microspheres, we used 10^–10^ dilution to finally isolate ureolytic bacteria for genomic analysis.

Culturing time was another important factor influencing the success to isolate single strains with embedding in agarose microspheres. As the incubation time increased from 12 to 72 h, the percentage of agarose microspheres that had bacteria increased (Supplementary Fig. 1 E), while that of agarose microspheres that had single strains decreased (Supplementary Fig. 1 F). To maximize the isolation of single strains, we selected 24 h incubation.

To assess the entry of microbial metabolites into the dialysis bags by diffusion so that the rumen microbes outside of the dialysis bags in the rumen-simulating system can provide nutrients and growth factors to the bacteria embedded in agarose microspheres inside the dialysis bags, we analyzed the metabolites both inside and outside of dialysis bags using untargeted metabolomics with LC/MS. In total, we detected 4224 metabolites in the dialysis bags and the simulated rumen fermentation system, of which 109 could be identified (Supplementary Fig. 2 A). All these metabolites were detected inside and outside the dialysis bags by 6 h after the dialysis bags were placed into the simulated fermentation system (Supplementary Fig. 2B). As shown by a clustering heatmap, the metabolite profiles both inside and outside of the dialysis bags varied over time, but they were similar at each time (Supplementary Fig. 2 B). The correlation coefficient of metabolite profiles between inside and outside the dialysis bags reached 0.96 ~ 0.98 at each incubation time (Supplementary Fig. 2 C).

### Genomic taxonomy of ureolytic bacteria isolates

**Fig. 2:**
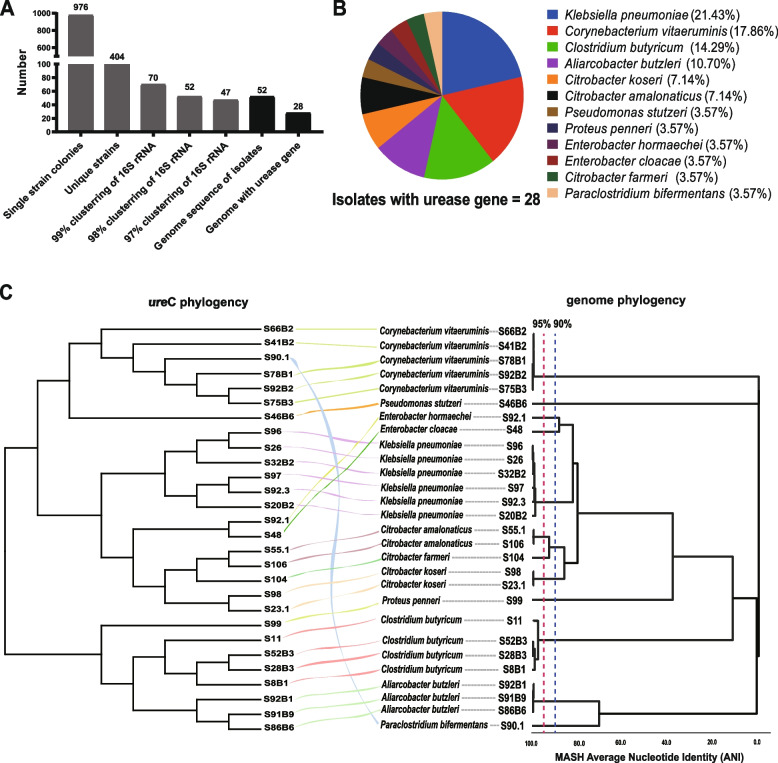
Classification and phylogeny of isolated ureolytic bacteria. **A** Number of colonies and strains during enrichment and isolation. **B** Species of the ureolytic isolates. **C** Phylogeny trees based on *ure*C gene and genomes of the isolated ureolytic bacteria

We obtained a total of 976 isolates of bacteria, of which 404 had a unique 16S rRNA gene sequence and represented 52 clusters of 16S rRNA gene identity < 98% (Fig. 2 A). Whole-genome sequencing and gene annotation identified 28 strains carrying urease genes (Fig. 2A, B). The genome features of each ureolytic isolate are listed in Supplementary Table 1. Their genomes ranged from 1.8 to 7 Mbp and were > 90% complete with < 7% contamination. Based on the average nucleotide identity (ANI) cutoff values for species (95%) and genus (90%) [31], the ureolytic genomes were grouped into 12 species in 11 genera (Fig. 2B). Taxonomic classification using GTDB-TK identified these 12 bacterial species as *Pseudomonas stutzeri* (1 strain), *Proteus penneri* (1), *Klebsiella pneumoniae* (6), *Enterobacter hormaechei* (1), *E. cloacae* (1), *Citrobacter koseri* (2), *C. farmeri* (1), *C. amalonaticus* (2), *Paraclostridium bifermentans* (1), *Clostridium butyricum* (4), *Aliarcobacter butzleri* (3), and *Corynebacterium vitaeruminis* (5) (Fig. 2 C, Supplementary Table 1). The ureolytic species accounted for 48% of all the ruminal ureolytic bacterial species whose genomes were sequenced. The phylogenetic tree topologies based on genome and *ure*C gene sequences were similar for all the ureolytic isolates except for strain S90.1 (*P. bifermentans*), S92.1 (*E. hormaechei*), and S48 (*E. cloacae*), which indicates the *ure*C gene could be used as a phylogenic marker gene for the taxonomy of ureolytic bacteria (Fig. 2B).

### Evaluation of ureolytic isolates compared to the previous studies

**Fig. 3:**
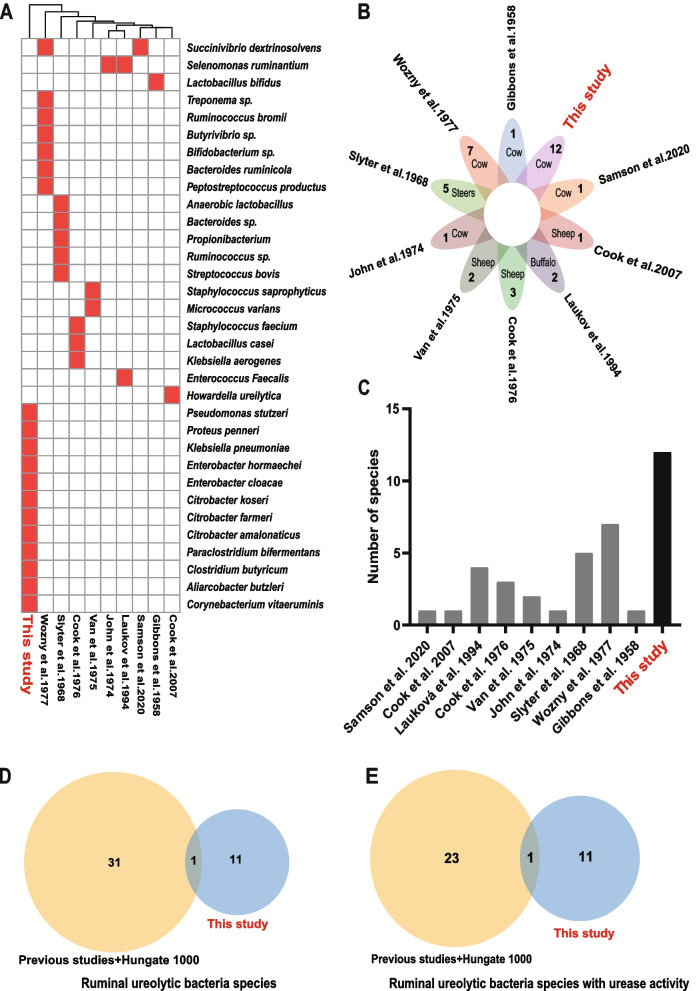
Comparison of the new ureolytic bacteria with those reported in the literature.** A** A heatmap of ureolytic bacterial species from this study and previous studies. **B** A Venn diagram of ureolytic bacterial species from this study and previous studies. **C** The number of ureolytic bacterial species isolated from this study and previous studies. **D** A Venn gram comparing the ureolytic bacteria of this study with those of previous studies plus Hungate1000 project. **E** A Venn diagram comparing the number of ureolytic bacteria isolates with ureolytic activities between this study and previous studies plus Hungate1000 project

All the 12 ureolytic species isolated in this study differed from the ureolytic species isolated from the nine previous studies (Fig. 3A, B). Compared with all the previous studies, the current study expanded the number of isolated ureolytic bacteria (Fig. 3C). In the rumen Hungate1000 project, 17 ureolytic bacteria species carry urease genes, of which three (1.7%) species had ureolytic activity as inferred from the BacDive database (Supplementary Table 2). A total of 35 ureolytic bacteria species have been isolated from the rumen in 10 previous studies, of which 24 species were inferred to have ureolytic activity according to the BacDive database (Supplementary Table 2). We tested the 12 new isolated species for their activity to hydrolyze urea, and all exhibited ureolytic activity (Fig. 5B). The current study increased the number of species carrying urease genes by 34.38% and the number of species with verified ureolytic activity by 45.83% (Fig. 3D, E).

### Occurrence and distribution of new ureolytic bacteria

**Fig. 4:**
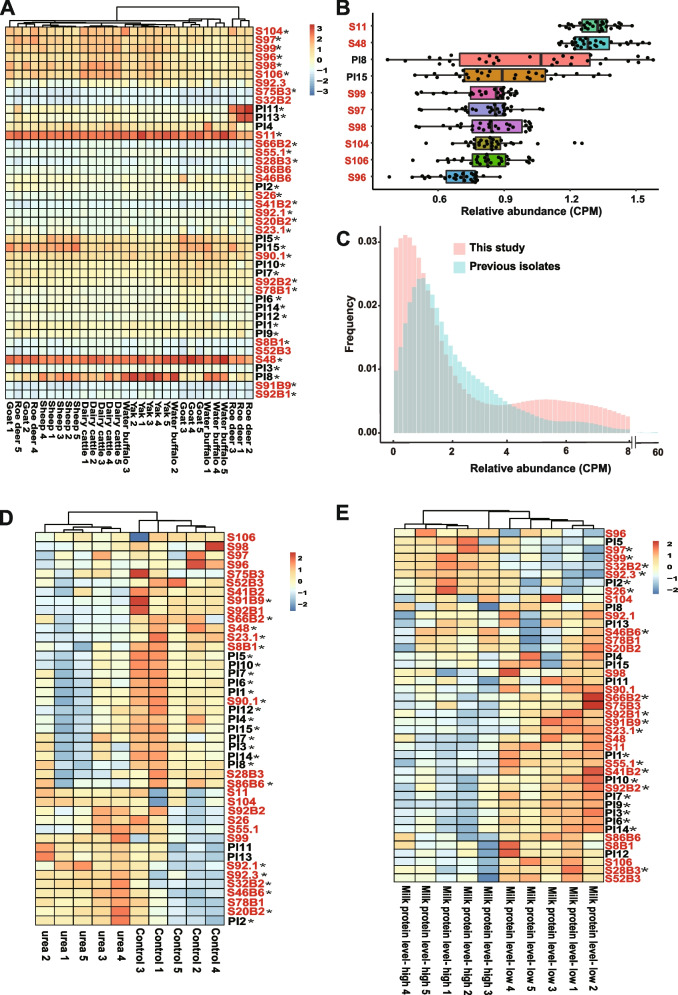
Relative abundance, prevalence, and contribution of ureolytic isolates.** A** A heatmap showing the relative abundance (copies per million, CPM) of ureolytic bacteria in the rumen of six different species of ruminants. **B** The 10 most abundant ureolytic bacteria in different ruminant species. **C** The prevalence of ureolytic bacteria with different relative abundance from this study and previous studies, **D** A heatmap showing the relative abundance of ureolytic bacteria in the rumen of goats supplemented with urea (urea) or without urea (control). **E** A heatmap of relative abundance of ureolytic bacteria in dairy cattle with high or low milk protein levels. Asterisks (*) indicate a significant difference. Statistical significance was tested by nonparametric Wilcoxon rank-sum test

We assessed the occurrence of the new ureolytic bacteria in the rumen of different species of wild and domesticated ruminants, including goats, roe deer, sheep, dairy cattle, yaks, and water buffalo by mapping the genome sequences of each isolated ureolytic bacterium to the metagenomes recovered therefrom. The relative abundance of the ureolytic bacteria was assessed as a mapping rate in the rumen metagenomes of the above ruminant species and with respect to urea feeding and milk quality. All the new ureolytic bacteria were found in goats, roe deer, sheep, dairy cattle, yaks, and water buffalo (Fig. 4A). The relative abundances of individual ureolytic bacteria varied in different ruminant species. The samples from the same ruminant species were clustered together based on the relative abundance of the isolated ureolytic bacteria, except for roe deer, water buffalo, and goat. *P. penneri*, *K. pneumoniae*, *C. koseri*, *C. farmer*, and *C. amalonaticus* were more abundant in dairy cattle than in other ruminant species (Fig. 4 A, Supplementary Table 1). Of the 10 isolates with a high relative abundance, 8 were isolated from this study (Fig. 4B). Compared with the previous studies, the current study isolated ureolytic bacteria that appeared to be at very low abundance (Fig. 4C). We found that urea supplement in feeds decreased the relative abundance of the isolated strains of *A. butzleri*,* C. vitaeruminis*,* E. cloacae*,* C. koseri*,* C. butyricum*, and *P. bifermentans*, but increased that of the strains of *E. hormaechei*,* K. pneumoniae*, and *P. stutzeri* in the rumen of sheep (Fig. 4D). In addition, 22 (51% of all) ureolytic bacteria were significantly associated with milk protein levels in dairy cattle (Fig. 4E), and 64% of these ureolytic bacteria were isolated in this study. The dairy cows with a high level of milk protein had a high abundance of isolates of *P. penneri*,* K. pneumoniae*,* P. stutzeri*, and low abundance of the strains of *C. vitaeruminis*, *A. butzleri*,* C. koseri*,* C. amalonaticus*, and *C. butyricum*.

### Unique gene families and glycoside hydrolases of the ureolytic isolates

**Fig. 5:**
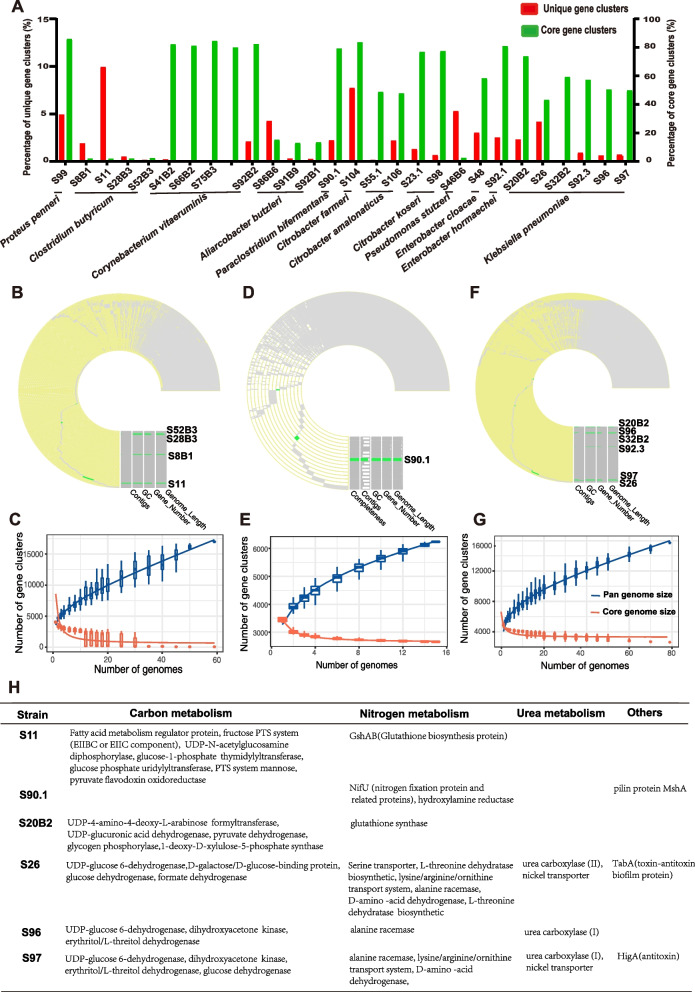
Pan-genome analysis of ureolytic bacteria.** A** The percentage of unique and core gene clusters of each ureolytic bacterial strain isolated in this study. **B** A circular diagram of the core and unique gene clusters of ureolytic bacteria classified to *Clostridium butyricum*. **C** Pan-genome profiles of ureolytic bacteria of *Clostridium butyricum*. Pan- and core-genome sizes were predicted from all the strains of individual species. **D** A circular diagram of the core and unique gene clusters of ureolytic bacteria classified to *Paraclostridium bifermentans*. **E** Pan-genome profiles of ureolytic bacteria of *Paraclostridium bifermentans*. **F** A circular diagram of the core and unique gene clusters of ureolytic bacteria classified to *Klebsiella pneumoniae*. **G** Pan-genome profiles of ureolytic bacteria of *Klebsiella pneumoniae*. **H** The function of unique genes in the species of *Clostridium butyricum*, *Paraclostridium bifermentans*, and *Klebsiella pneumoniae*

We compared the genomes of the 28 ureolytic isolates with those of some ureolytic bacteria isolated previously by other researchers with respect to ureases and glycoside hydrolases (GHs). All the new isolates had unique gene families, ranging from 0.07 to 9.87% of all gene families (Fig. 5 A, Supplementary Fig. 3). The pan-genome size for each species increased but did not reach a plateau as more genomes were added, which indicates an open pan-genome status for those species (Fig. 5C, E, and G, Supplementary Fig. 4). According to the number of unique gene families and their high annotation rate, three species (*C. butyricum*, *P. bifermentans*,* K. pneumoniae*) were selected to examine the distribution of the unique gene families. We found 65, 2662, and 2637 core gene families from the genomes of *C. butyricum*, *P. bifermentans*, and *K. pneumoniae*, respectively (Fig. 5B, D, and F)*.* For *C. butyricum*, 57 strains were found, including four strains isolated in this study. These four new strains had 3 to 420 unique gene families, but strain S11 was the predominant one (Fig. 5B). *P. bifermentans* had 16 strains, and only one strain (S90.1) was isolated in the current study. This new strain had 73 unique gene families (Fig. 5D). *K. pneumoniae* was represented by the most strains (79 to be exact), and six strains were isolated in this study, and they had 3–254 unique gene families (Fig. 5F). Most of the unique gene families of these three species are involved in carbon and nitrogen metabolism (Fig. 5H). The unique gene families of strain S11 (*C. butyricum*) were involved in fatty acid metabolism, carbohydrate metabolism, and glutathione biosynthesis. Strain S90.1 (*P. bifermentans*) had unique gene families involved in nitrogen fixation and pilin synthesis. The two new isolates of *K. pneumoniae* had the unique functions of L-arabinose, formate, L-threitol, D-galactose and D-xylulose metabolism, and glutathione, valine, serine, ornithine, L-threonine, alanine, lysine, and arginine metabolism. Interestingly, strains S26, S96, and S97 of *K. pneumoniae* were found to have a unique gene family involved in urea metabolism, urea carboxylase, which can form a hydrolysis system with allophanate hydrolase*.*

The rumen ecosystem uses polysaccharides as the major carbon and energy sources, and thus, we examined the genomes of the 28 ureolytic isolates for the occurrence of GHs using dbCAN and the CAZy database. We identified GHs involved in the hydrolysis of different types of polysaccharides including starch, hemicellulose, cellulose, and oligosaccharide (Supplementary Table 3). Different strains of the same species had similar types and families of GHs. The stains of *C. amalonaticus*, *C. farmeri*, *C. koseri*, *C. butyricum*, *E. cloacae*, *E. hormaechei*, and *K. pneumoniae* contained both amylases, cellulases, hemicellulases, and oligosaccharide-degrading enzymes, which indicates that they can utilize a wide range of polysaccharides and contribute to feed digestion.

### Urease gene cluster diversity, urease activity, and key amino acid residues of the UreC

**Fig. 6:**
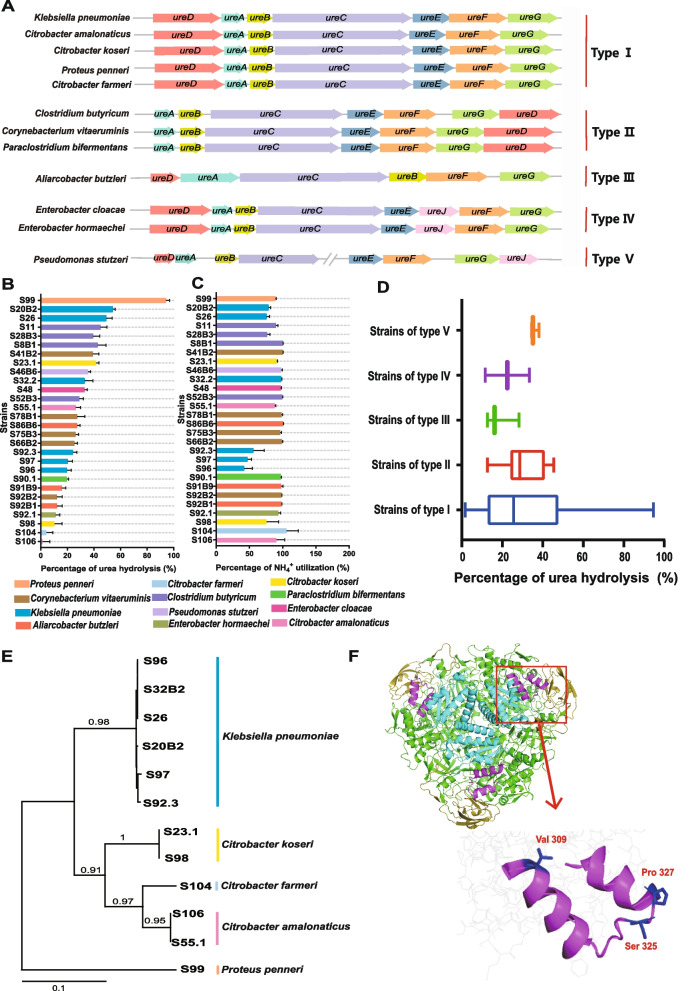
Urease gene clusters and urease hydrolysis activities of the isolated bacteria.** A** Schematics of urease gene clusters of the ureolytic bacteria isolated in this study. **B** Urea hydrolysis rates of each ureolytic isolate (*n* = 3). **C** Urea-N utilization rates of each ureolytic isolate (*n* = 3). **D** Box plots of urea hydrolysis rates of urease gene cluster types. **E** A phylogeny tree based on ammonia acid sequence of UreC of the isolated ureolytic bacteria that carry type I urease gene cluster. **F** The homology model of urease of S99. Subunits are indicated by different colors, in which the trimer of alpha subunits (UreC) is depicted as green, the beta subunits (UreB) as cyan, the gamma subunits (UreA) as yellow and the flap regain as red. The flap regain of UreC is magnified, in which the varied AA residues (Val^309^, Ser^325^, Pro^327^) were labeled in blue

Urease genes are typically organized as clusters in the genomes of ureolytic bacteria, and they include three structural genes (*ure*A, *ure*B, *ure*C*)* and a few of five accessory genes. Among the urease-carrying isolates from the current study, we found five types of urease gene clusters (Fig. 6A, Supplementary Fig. 5). These clusters had the three structural genes, but they differed in the presence or order of the accessory genes. Several open reading frames (ORFs) were found between *ure*C and *ure*E in type V cluster. Compared with the other cluster types, type IV and type V clusters had *ure*J, which encodes a nickel-containing transmembrane transporter.

All ureolytic bacterial isolates had urease activity though they differed in activities of urea hydrolysis or utilization. Hydrolysis of the urea added to the culture medium (0.05 g/100 ml) by the isolates ranged from 2 to 95%, with strain S99 (*P. penneri*) having the greatest, while strain S106 (*C. amalonaticus*) the lowest urea hydrolysis (Fig. 6B). Urea hydrolytic activities differed among some strains of the same species, such as strains of *K. pneumoniae*, *C. koseri*, *C. amalonaticus*, and *A. butzleri*. Strains of *K. pneumoniae*, *C. koseri*, *C. amalonaticus*, and *A. butzleri* hydrolyzed 20–54%, 11–42%, 2–27%, and 13–28%, respectively, of the urea added to the medium. Urea hydrolytic activities per unit of culture optical density mirrored the total hydrolysis activities (Supplementary Fig. 6A). Interestingly, their ability to utilize the ammonia-N produced by urea-N did not coincide with that to hydrolyze urea, either total or per unit of culture optical density (Fig. 6C, Supplementary Fig. 6B). In particular, strain S90.1 (*P. bifermentans*) had the highest ammonia-N utilization produced from urea per biomass, but it had lower urea hydrolysis efficiency.

The urea hydrolysis rates did not differ among the different urease gene clusters due to large variations within most of the clusters (Fig. 6D). The strains that had urease gene cluster I had the highest variation of urea hydrolysis rate. We aligned the amino acid (AA) sequences of UreC, which carries the active center of urease, among the strains carrying this cluster to reveal the potential mechanism of regulating strain’s urease activity. Strains of the same species had nearly identical UreC sequences (99 ~ 100%), while strains across different species shared lower UreC sequence similarity (91 ~ 98%), with the UreC of strain S99 being only 71 ~ 73% similar to the UreC sequences of the other strains/species (Supplementary Table 4). On the phylogenetic tree of UreC, strain S99 formed its only branch (Fig. 6E). We examined the UreC sequence alignment and compared the key catalytic residues (His^134^, His^136^, Lys^217^, His^246^, His^272^, Asp^360^) in the UreC active center [32] of strain S99 and the other isolates that carried cluster I (Supplementary Fig. 7) and found that the key catalytic residues in UreC were conserved. However, the flap region that forms a flexible loop covering the active center of strain S99 had three mutated AAs (Val^309^, Ser^325^, Pro^327^) compared to other isolates that carried cluster type I (Fig. 6F, Supplementary Fig. 7). The Ser^325^ and Pro^327^ were located in the turn region that controls the opening or closing of the active center. These two AA residues may explain the high urease activity observed for S99.

## Discussion

A major hurdle to fully revealing the functional activity of bacteria is the difficulty to isolate and culture individual strains from complex communities. It is more challenging to isolate bacteria that are present at low abundance and difficult to enrich with a selective carbon or energy source, such as ureolytic bacteria from the rumen because fast-growing, abundant bacteria often inhibit the growth of slow-growing ones [33]. The latter is also “masked” by the former, which results in biased isolation of fast growers. Because of this, there are only 32 published ureolytic bacteria species isolated from the rumen of different ruminants using the traditional plating method although nearly 600 ureolytic species were found according to *ure*C gene sequences in the rumen of cattle [5]. Therefore, tailored methodology for the enrichment proper enrichment and selection of bacteria of interest are needed to effectively isolate a given functional group of bacteria.

In the present study, we established an integrated low-cost method compared with Raman-activated cell sorting [34] and flow cytometry [35] that allowed for the enrichment and isolation of rumen ureolytic bacteria. This method allowed for the enrichment of ureolytic bacteria, embedding and then allowing the growth of single bacterial cells in agarose microspheres, selection of those that carry urease genes (the bacteria of our interest), and incubation of the selected urease gene-carrying individual strains in dialysis bags placed in rumen-simulating conditions. With the integrated method and equipment commonly available in a microbiology laboratory, this integrated method allowed us successfully obtain 404 bacterial isolates. Genome sequencing of 52 isolates each representing a group with > 98% 16S rRNA gene sequence identity helped identify 28 isolates that carry urease genes, and some of the isolated bacteria appeared to be at low abundance in the rumen. Compared to those that represent the “global” rumen bacterial genus [36], the present study newly isolated and identified bacteria of *Pseudomonas*, *Proteus*,* Citrobacter*, and* Aliarcobacter*. This indicates that isolating individual microbes can help better study the integral rumen microbiome. Moreover, the current study obtained more ureolytic bacterial isolates than any previous published studies [10–18], and all the newly isolated ureolytic bacteria represent previously uncultured bacteria in the rumen. Analysis of the abundance of the new ureolytic bacteria across multiple ruminant species showed that some of the isolated ureolytic strains were abundant while others were minor members of the rumen microbiome. This suggests that this integrated method can help isolate bacteria at low abundance, some of which can be important to the function of microbial ecosystems [37, 38]. It should be noted that the geographical variation and diets type can influence the microbial composition or abundance, which may also contribute to the possibility of isolation of novel species or strains [36, 39]. It should be noted that we only sequenced the genomes of 52 isolates. More ureolytic bacteria will be expected if more strains are sequenced in future studies.

Embedding diluted bacteria in microspheres to aid their isolation has been used in isolating bacteria from other environments [40–44]. Here, we used agarose microspheres that are large enough to be picked and transferred manually without any specialty tools. The relatively large agarose microspheres also allowed for sufficient microbial growth and biomass for DNA extraction and genome sequencing. The use of dialysis bags placed in habitat-simulating conditions for in situ incubation probably enabled the growth of bacteria that require cross-feeding with other bacteria. To the best of our knowledge, this is the first study that isolates microbes from the rumen using in situ incubation though other researchers have used it to isolate bacteria from other environments [45–47]. A method called ichip combining bacterial dilution and in situ incubation can increase the recovery of microbes by 5 to 300 fold [45], but it uses 96-well plates during in situ incubation, which are much larger and takes up space compared to the microsphere we used. It should be noted that we optimized the dilution rates and incubation times to maximize the isolation of ureolytic bacteria. However, different dilution rates and incubation time will probably be needed to isolate different bacteria from different environments. In addition, manual picking and transferring of individual agarose microspheres limit the throughput of the method. Automated systems, such as the COPAS flow-sorting system [48], can be used to increase the throughput and number of bacterial isolates.

All the ureolytic bacteria isolated in this study were found in goats, roe deer, sheep, dairy cattle, yaks, and water buffalo, but their occurrence varied among the different ruminant species. These isolates may be among the core ruminal bacteria and have potentially co-evolved with the ruminants. Using the rumen metagenomic data from sheep [49] and lactating cows [50], we explored the roles of these isolates in urea metabolism in the rumen of sheep and milk protein synthesis of dairy cattle. We found that dietary urea supplementation decreased the relative abundance of the ureolytic isolates of *C. butyricum*, *A. butzleri*, *C. vitaeruminis*,* E. cloacae*,* C. koseri*, and *P. bifermentans* but increased that of the strains of *E. hormaechei*, *K. pneumoniae*, and *P. stutzeri* in sheep. The decrease in *C. butyricum* concurs with the decreased abundance of the genus *C.* reported in the metagenomic data [49]. However, all the ureolytic bacteria that were increased by the urea supplementation were not found in the metagenomic data. In the lactating dairy cows, high levels of milk protein corresponded with high abundance of the ureolytic bacteria of *P. penneri*, *K. pneumoniae*, and *P. stutzeri* but a low abundance of those of *C. vitaeruminis*, *A. butzleri*, *C. koseri*, *C. amalonaticus*, and *C. butyricum*. These species were not to be associated with milk protein levels in the metagenomic study [50]. These results suggest that isolation and quantification of genome-sequenced bacteria may help better understand the association between specific rumen microbes and lactation performance in dairy cows. Future research is needed to further examine the association between ureolytic bacteria and milk protein levels in dairy cows.

A pangenome analysis revealed some interesting functional genes unique to some of the ureolytic isolates. Compared with the other ureolytic stains of *P. bifermentans*, strain S90.1 contained unique nitrogen fixation genes that encode enzymes converting N_2_ to ammonia. Even though nitrogen fixation was found in the rumen nearly 50 years [51], little is known about the diversity of nitrogen-fixing microorganisms and their nitrogen fixation genes. Strain S90.1 of *P. bifermentans* can be used as a type strain to study nitrogen fixation in the rumen. Also interestingly, we found a unique gene encoding urea carboxylase in strains S96, S97, and S26 of *K. pneumoniae*. As a part of urea amidolyase, urea carboxylase converts urea to allophanate, which is then converted to ammonia by allophanate hydrolase [52]. Here, we found the two types of genes in the same ureolytic bacteria of *K. pneumoniae*. The contribution of these two types of enzymes to urea hydrolysis needs further studies. In addition, the isolating strains in this study contained both core genes encoding amylases, cellulases, hemicellulases, oligosaccharide-degrading enzymes, and some unique genes encoding proteins involved in carbohydrate metabolism, which indicates that they can utilize a wide range of polysaccharides and contribute to balancing dietary energy and nitrogen [53, 54] and high urea-N utilization efficiency.

There is limited information about the urease gene structure among rumen ureolytic microbes. Here, we found five types of urease gene clusters across the isolates. Types I and II are common and have been reported in the literature, but not the other three. The presence of *ure*J, a nickel transmembrane transporter [55], in types IV and V is of interest. Urease is a nickel-dependent enzyme. The recruitment of *ure*J in the urease gene cluster coordinates nickel transfer to apo-urease. Comparison of urea hydrolysis activities and the type of urease gene clusters among the isolates showed no correlation between them. It is interesting to note, however, two mutated AAs (Ser^325^ and Pro^327^) in the flap turn region of UreC of strain S99 corresponded to its highest urea hydrolysis activity. The flap is thought to act as a gate for the substrate [56, 57]. The helix-turn-helix (from residues 307 to 337) of the flap that covers the active site cavity is highly mobile, and it allows urea extensive access to the active site, and the AAs in the turn region control the movement of the flap [32]. Therefore, these two AA residues potentially play important roles in the urease activity of strain S99, but this hypothesis needs experimental verification. In addition, strain S99 can be a target to inhibit the urease activity of ruminal microbes to improve rea utilization efficiency in ruminants.

## Conclusion

In this study, we isolated ureolytic bacteria from the rumen using a targeted method, urease gene (*ure*C)-guided enrichment plus in situ microsphere cultivation. We isolated and characterized diverse ureolytic bacteria with demonstrated urease activity, and many of the new isolates have not been cultured previously from the rumen. Some of the new isolates may play important roles in nitrogen, especially urea and metabolism. These isolates can be used as model bacteria to further understand nitrogen metabolism, in particular urea metabolism, for improved urea utilization. The new methodology will also help isolate uncultured microbes of interest in the other environment to better bridge the knowledge gap between genotypes and phenotypes of uncultured bacteria.

## Methods

### Rumen microbiota sampling and medium preparation

Rumen content samples were collected from three cannulated Holstein dairy cows (body weight of 550 ± 50 kg) for medium preparation and as the source of ureolytic bacteria. All the procedures involving the care and management of dairy cows were approved by the Animal Care and Use Committee for Livestock of the Institute of Animal Sciences, Chinese Academy of Agricultural Sciences (protocol no.: IAS201914). The dairy cows consumed a typical total mixed ration (DM base) consisting of 36% corn silage, 23% corn powder, 7.9% soybean meal, 8.7% soybean hulls, 8.3% barley, 9.0% oat grass, 4.0% alfalfa hay, 0.8%, CaHPO_4_, 0.5% NaHCO_3_, 0.2% NaCl, 0.2% CaCO_3_, 0.1% C_5_H_14_ClNO, 0.1% calcium fatty acid, 0.1% double beneficial element, 0.1% rhodamine, and 1% urea. We collected rumen content samples from the three cows before morning feeding and filtered them through four layers of cheesecloth. Immediately, the filtered rumen fluid samples were pooled (equal volume), and the composite rumen fluid sample was injected into an anaerobic bottle containing an equal volume of sterile glycerol (15%, v/v) with a syringe and stored at − 80℃ to help maintain bacterial viability until use. Another aliquot of the three filtered rumen fluid samples was pooled (equal volume) and transferred to a bottle and stored at − 80℃ for preparation of urea medium and rumen-simulating conditions (detailed later).

The urea medium for enrichment was preparation anaerobic. Specifically, the medium (100 ml) contained 5 ml of clarified rumen fluid, 0.05 g urea, 0.05 g glucose, 0.05 g cellulose, 15 ml solution 4 (3 g/l K_2_HPO_4_), 15 ml solution 5 (0.6 g/l CaCl_2_, 3 g/l KH_2_PO_4_, 6 g/l NaCl, 0.6 g/l MgSO_4_·7H_2_O), 0.1 ml Pfennig trace element (300 mg/L H_3_BO_3_, 100 mg/L ZnSO_4_·7H_2_O, 30 mg/l MnCl_2_·4H_2_O, 20 mg/l CoCl_2_·6H_2_O, 30 mg/l Na_2_MoO_4_·2H_2_O, 10 mg/l Na_2_SeO_3_, 20 mg/l NiCl_2_, 10 mg/l CuCl_2_·2H_2_O, 150 mg/l FeCl_2_·4H_2_O), 5 ml hemin (0.05 mg/ml), 0.1 ml resazurin (0.1%), 0.31 ml volatile fatty acid (VFA) mix (17 ml/l acetic, 6 ml/l propionic, 4 ml/l *n*-butyric, 1 ml/l *n*-valeric, 1 ml/l isovaleric, 1 ml/l isobutyric, 1 ml/l 2-methyl butyric acids), 0.8 g NaHCO_3_, 0.05 g L-cysteine HCl, and 59.49 ml ddH_2_O. We prepared one dilution solution and made it anaerobic to dilute the composite inoculum. It contained (1000 ml) 38 ml solution 2 (6 g/l K_2_HPO_4_), 38 ml solution 3 (1.6 g/l CaCl_2_·2H_2_O, 6 g/l KH_2_PO_4_, 12 g/l NaCl, 6 g/l (NH_4_)_2_SO_4_, 2.5 g/l MgSO_4_·7H_2_O), 8 g Na_2_CO_3_, 1 ml resazurin (0.1%), and 0.5 g L-cysteine HCl [11].

### Urease gene-guided enrichment of ureolytic bacteria of the rumen

We serially diluted (tenfold, 10^0^ to 10^–7^) the pooled rumen fluid inoculum preserved in glycerol at − 80℃ in the anaerobic diluent solution inside an anaerobic chamber with an atmosphere of 85% N_2_, 10% CO_2_, and 5% H_2_ (all the experimental procedures involving live bacteria were performed in the anaerobic chamber). We then mixed 200 μl of each diluted inoculum with 20 ml of the medium. After thorough mixing, 200 μl was dispensed into each well of one 96-well plate (one plate for each dilution). We sealed all plates with microplate sealing film and incubated the plates at 39 °C for 48 h. After incubation, we extracted bacterial DNA from each well (all the wells, separately) using the alkaline lysis method [33]. Briefly, 10 μl of bacterial culture from each well was combined with 16.6 μl of lysis buffer (25 mM NaOH, 0.2 mM Na_2_-EDTA, pH 12) and incubated at 95 °C for 30 min, followed by neutralization with the addition of 16.6 μl of Tris–HCl (40 mM, pH 7.5). We screened the extracted DNA from all the wells for the presence of *ure*C gene with the *ure*C-F and *ure*C-R primers and PCR [6]. The *ure*C-positive cultures of the highest dilution were selected to isolate ureolytic bacteria to minimize contamination with non-ureolytic bacteria. The remaining cultures of the *ure*C-positive wells of the selected dilution (10^–4^, see Results) were combined and served as the enriched ureolytic bacteria.

### Bacterial embedding in agarose microspheres and in situ-simulated cultivation

We isolated ureolytic bacteria using agarose embedding and then incubation in dialysis bags (detailed below) in a rumen-simulating “in situ” system. Briefly, in the anaerobic chamber we combined 1 L of the glycerol frozen rumen fluid, 1 L of anaerobic diluent solution, 30 g of a total mixed ration in an anaerobic bottle to create a rumen-simulating in situ system. Dialysis bags (1000 kDa) (Sigma Chemical Co., St. Louis, MO, USA) each containing 100 ml of anaerobic solution were placed into the rumen-simulating bottle and inoculated at 39 °C. Then, 5 ml of sample each was collected from inside of each dialysis bag and outside the dialysis bags at 6, 12, 24, and 72 h (3 bags at each time point) and subjected to metabolomic analysis using LC–MS/MS to assess the entry of metabolites into the dialysis bags over time. Briefly, metabolites were extracted, vacuum dried following the collection of the top phase, and dissolved in 100 μl of 1% acetonitrile [58]. The metabolites were resolved using an LC–MS/MS system consisting of an Agilent 1290 II (Agilent Technologies, Germany) and a 5600 Triple TOF Plus (AB Sciex, Singapore). The peak area, mass-to-charge ratio, and retention times of the original first-level MS data were extracted using MarkerView 1.3 [59] (AB S CIEX, Concord, ON, Canada) to generate a two-dimensional datum matrix (filter isotope peak). Secondary MS data were extracted from original MS files with PeakView 2.2 [60] (AB S CIex, Concord, ON, Canada) and compared with the HMDB [61] and METLIN [62] databases to identify the metabolites in the samples. The metabolite profiles were subjected to principal component analysis (PCA) and cluster analysis using MetaboAnalyst 5.0 [63].

We serially diluted (to-fold, 10^0^ to 10^–10^) the ureolytic bacteria from the 10^–4^ dilution of the enrichment in the anaerobic diluent solution. After thoroughly mixing 1 ml of each dilution with 9 ml of preheated (40 ℃) urea medium containing 1.5% (w/v) melted low-melting agarose (Shanghai yuan ye Bio-Technology Co., Ltd., Shanghai, China), we embedded the bacteria in agarose microspheres by dripping agarose droplets into cold mineral oil through a 10-ml syringe with a 0.45 mm needle and a Programmable Syringe Pump (New Era Pump Systems, New York, USA) at a constant speed of 4 ml/min to ensure uniformed microsphere size. We washed the agarose microspheres thrice with anaerobic sterile water to remove the mineral oil. The average diameters of agarose microspheres were determined microscopically based on 300 microspheres.

All the agarose microspheres from each of the dilutions were placed into one dialysis bag (1000 kDa). The dialysis bags of all but 10^–10^ dilutions were incubated in the simulating in situ rumen fermentation system for 24 h at 39 ℃. The dialysis bags of the 10^–10^ dilution were incubated the same as the above but subsampled at 12, 24, 48, and 72 h of incubation. At each time point and for each dilution, 192 agarose microspheres were individually placed into the well of two sterile 96-well plates. The microspheres were crushed with sterile nail heads followed by the addition of 200 μl urea medium per well and incubation at 39 °C for 24 h. We then collected 10 μl from each well for DNA extraction (see above) and PCR amplification of the 16S rRNA gene using universal bacterial primers 27-F and 1492-R [64]. The amplicons were purified and then sequenced using primer 27-F on an ABI 3730 system (ThermoFisher Scientific, USA) [65]. The sequences were visually checked using BioEdit [66] to determine if the microspheres had single strains (no double peak for any nucleotide). The 16S rRNA gene sequences of all the single strains were clustered at 100%, 99%, 98%, and 97% using Mothur [67]. The strains that differed in their 16S rRNA gene by > 2% were selected for genomic sequencing, and their remaining cultures served as the inoculate to prepare a larger volume of cultures for genomic DNA extraction.

### Genome sequencing and bioinformatic analyses

In total, 52 microsphere cultures that were assumed to represent different (16S rRNA genes sequence identify < 98%) single strains were chosen for genomic analysis. Briefly, each microsphere culture (0.1 ml) was inoculated into 10-ml urea medium (1% inoculum) and then incubated at 39 °C for 24 h. We extracted genomic DNA from 2 ml of each culture using the cetyltrimethylammonium bromide (CTAB) method plus bead beating [5]. DNA quality was evaluated using agarose (1%) gel electrophoresis, and DNA concentration was measured by a Qubit® 3.0 Fluorometer (Invitrogen, USA). Individual sequencing libraries were prepared for all the cultures using the NEB Next® Ultra™ DNA Library Prep Kit (Illumina, Inc., San Diego, USA) following the manufacturer’s instructions. The DNA libraries were paired-end (2 × 150 bp) sequenced on the NovaSeq 6000 system (Illumina). The raw sequence reads were subjected to adapter removal and quality filtering using trimmomatic [68] and fastqc [69], respectively. The clean reads were assembled using MetaHIT [70]. We checked the quality of the genomes with CheckM [71] and de-replicated the high-quality genomes (> 90% complete, < 10% contamination) using dRep at a threshold of 95% ANI over the default length [72, 73]. The genomes were classified using the Genome Taxonomy Database Tool kit (GTDB-Tk) [74]. Gene prediction and annotation were performed using Prokka [75] and eggNOG mapper [76], respectively. Predicted urease gene clusters were visualized using the gggenes package v0.4.0 [77] in R. Phylogenetic analysis of *ure*C genes was performed using MEGA X [78].

The genomes of 418 ruminal isolates sequenced in the Hungate1000 project were downloaded [19]. Then the genes were predicted and annotated with Prokka and eggNOG mapper. The classification of the genomes carrying urease genes obtained in the present study was compared with that of the urease-carrying strains of the Hungate1000 project and those reported in nine previous studies [10–18] to identify new ureolytic bacteria we obtained in the present study. We also experimentally determined the ureolytic activity of 28 *ure*C-positive isolates (see below) and compared their classification against those of the Hungate1000 strains with urease activity recorded in the BacDive metadatabase [79] and the ureolytic bacterial strains isolated from the rumen and shown to be ureolytic in the nine publications [10–18]. Heatmap and Venn diagram were generated using ImageGP [80] to show the comparison between our new isolates and the isolates that have been previously reported.

### Prevalence and occurrence of ureolytic bacteria

The genomes of the new ureolytic isolates and those of the Hungate1000 carrying ureases were combined as reference genomes. We then mapped the metagenomic datasets from three studies to the reference genomes to assess the prevalence of ureolytic bacteria that we isolated in the present study. Briefly, the largest rumen metagenomic dataset from six different species of ruminants (GenBank accession No.: SRR12529079) [81], the rumen metagenomic data of sheep supplemented with or without urea (SRR11784296) [49], and the rumen metagenomic data from two groups of dairy cows with low and high milk protein levels (PRJNA526070) [50] were downloaded. The relative abundance (copies per million, CPM) of the genomes of ureolytic isolates was determined with MetaWRAP [82] using the Quant_bins module. Heatmap, box plot, and histogram were generated using ImageGP [80] to compare the relative abundance of ureolytic bacteria that can be identified with the addition of the new ureolytic genomes. Statistical significance (*P* < 0.05) was tested with Wilcoxon rank-sum test. To identify the unique and core genes in each species, we did a pan-genome analysis using Panaroo at sequence 70% identity and 95% ratio [83]. The published genomes of 12 species were downloaded from the NCBI database (Supplementary Table 5). The identified genes of each isolate genome were further analyzed for glycoside hydrolases using dbCAN against the CAZy database [84].

### Ureolytic activity and key AAs sites of the bacterial isolates

All isolates with urease genes were cultured anaerobically in 10 ml of urea medium (containing 0.05 g urea per 100 ml) at 39 °C for 24 h (three replicates per isolate). Control (*n* = 3) was the same as the culture but with no bacterial inoculation. After the incubation, bacterial growth was measured as optical density at 600 nm. Two ml of each culture was mixed with 0.2 ml of 25% metaphosphoric acid and centrifugated at 12,000 × g for 5 min. The supernatant was collected for determination of NH_3_-N and urea-N using the Berthelot alkaline phenol-hypochlorite method [85] and the diacetyl monoxime method kit (Nanjing Jiancheng Co., Nanjing, China), respectively. Urea hydrolysis activity was calculated as the amount of urea decreased and the amount of ammonia increased in each culture over the 24 h incubation after subtracting the “abiotic” urea decrease and ammonia decrease in the control. The amounts of urea hydrolyzed and ammonia produced were also normalized per OD of each culture.

We aligned the UreC sequences of type I urease gene cluster to identify the AAs that might attribute to the high urea hydrolysis activity of strain S99 using Clustalw in BioEdit [66]. A phylogenetic tree of UreC was constructed using neighbor-joining method in MEGA X [78]. The three-dimensional structure of the urease structural protein complex (UreA, UreB and UreC) was modeled using the SWISS-MODEL server with default parameters [86] and visualized by PyMOL [87].

## Supplementary Information

**Additional file 1: Supplementary Table 1. **Summary of genome features of the ureolytic bacterial isolates.

**Additional file 2: Supplementary Table 2. **All ruminal ureolytic bacteria that have been reported in the literature.

**Additional file 3: Supplementary Table 3.** The major GH families identified in each isolate.

**Additional file 4: Supplementary Table 4. **The similarity of UreC amino acid sequences of the isolates with urease gene cluster type I.

**Additional file 5: Supplementary Table 5. **The ureolytic bacteria whose genome were used for pan-genome analysis.

**Additional file 6: Supplementary Fig. 1. **Optimization of isolation methodology of functional gene-guided enrichment plus in situ microsphere cultivation for ureolytic bacteria. A. A plot of ureC-positive wells vs. dilution; B. Distribution of agarose microsphere sizes (diameter); C. Percentage of agarose microspheres with embedded bacteria at different dilutions; D. Percentage of agarose microspheres with embedded single bacteria at different dilutions; E. Percentage of agarose microspheres with PCR-detected embedded bacteria with increasing incubation time; F. Percentage of agarose microsphere with PCR-detected single bacteria with increasing incubation time.

**Additional file 7: Supplementary Fig. 2. **Microbial metabolites inside and outside of the dialysis bags. A. The identified metabolites inside the dialysis bags; B. A heatmap of the metabolite profiles at each incubation time both inside and outside of the dialysis bags; C. Correlation of metabolite profiles between insides and outside the dialysis bags.

**Additional file 8: Supplementary Fig. 3. **Circular diagrams of the core and unique gene clusters of the ureolytic isolates of individual species.

**Additional file 9: Supplementary Fig. 4. **Pan-genome profiles of ureolytic isolates. Pan-genome (blue) and core-genome (red) sizes were predicted based on all the strains of individual species.

**Additional file 10: Supplementary Fig. 5. **Urease gene clusters identified among the ureolytic isolates.

**Additional file 11: Supplementary Fig. 6. **Urea hydrolysis rates (A) and urea-utilization rates of each ureolytic isolate per unit of culture optical density (OD, *n* = 3).

**Additional file 12: Supplementary Fig. 7. **Sequence alignment of UreC of type I urease gene cluster.

## Data Availability

The rumen metagenome data from different species of ruminants were from GenBank with No. SRR12529079. The rumen metagenome data of sheep supplemented with or without urea were from GenBank with No. SRR11784296. The rumen metagenome data from dairy cows with low and high milk protein levels were from GenBank with No. PRJNA526070. The complete genome sequences for the 28 new ureolytic isolates have been deposited in China National Microbiology Data Center (https://nmdc.cn/en) under the No. NMDC10018215. Ethics approval and consent to participate.

## Abbreviations

*ure*C: Urease gene
MPS: Microbial protein synthesis
ANI: Average nucleotide identity
GHs: Glycoside hydrolases
AA: Amino acid
PCA: Principal component analysis
CTAB: Cetyltrimethylammonium bromide
