# Supplementary figures and images for: Functional gene-guided enrichment plus in situ microsphere cultivation enables isolation of new crucial ureolytic bacteria from the rumen of cattle
Source: Microbiome. 2023 Apr 15;11:76. doi: 10.1186/s40168-023-01510-4 (PMC10105427; doi:10.1186/s40168-023-01510-4)

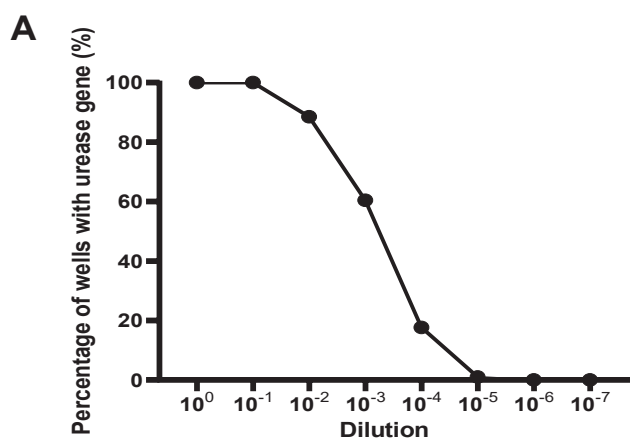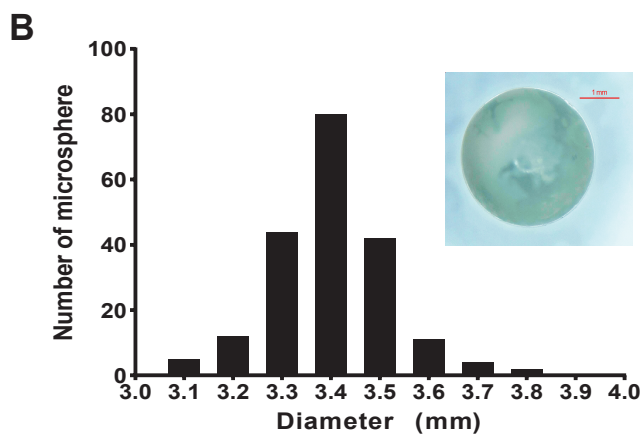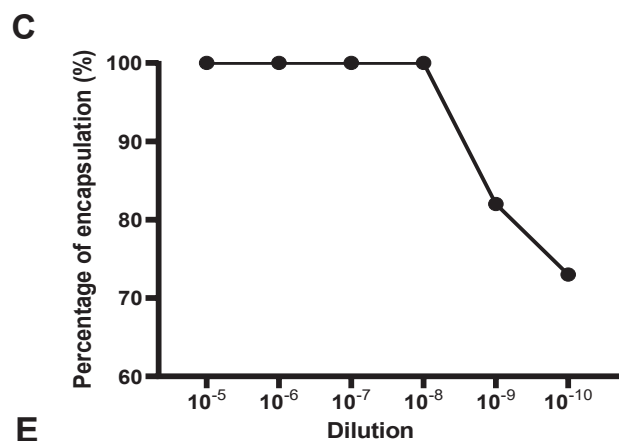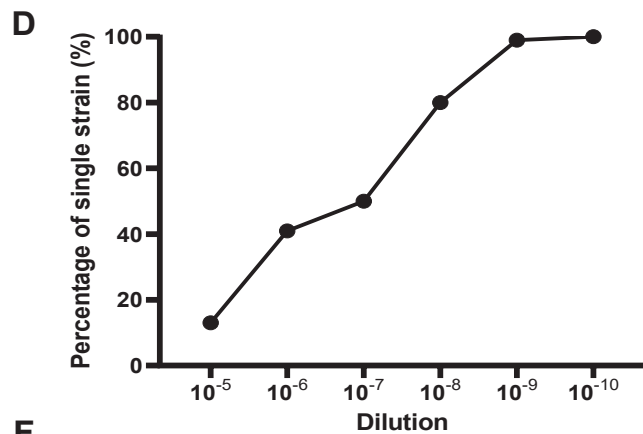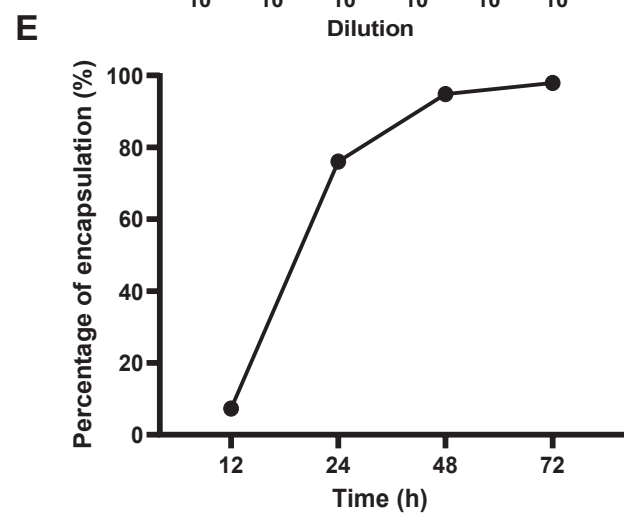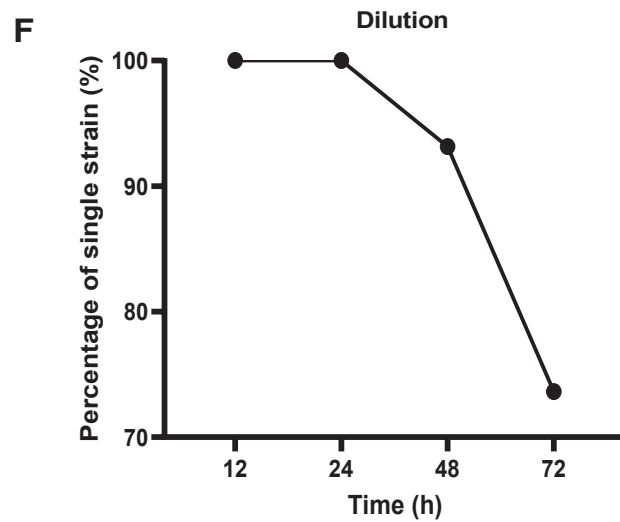

Supplement: Supplementary file 7 — Additional file 6: Supplementary Fig. 1. Optimization of isolation methodology of functional gene-guided enrichment plus in situ microsphere cultivation for ureolytic bacteria. A. A plot of ureC-positive wells vs. dilution; B. Distribution of agarose microsphere sizes (diameter); C. Percentage of agarose microspheres with embedded bacteria at different dilutions; D. Percentage of agarose microspheres with embedded single bacteria at different dilutions; E. Percentage of agarose microspheres with PCR-detected embedded bacteria with increasing incubation time; F. Percentage of agarose microsphere with PCR-detected single bacteria with increasing incubation time. [file 40168_2023_1510_MOESM6_ESM.pdf]

**A**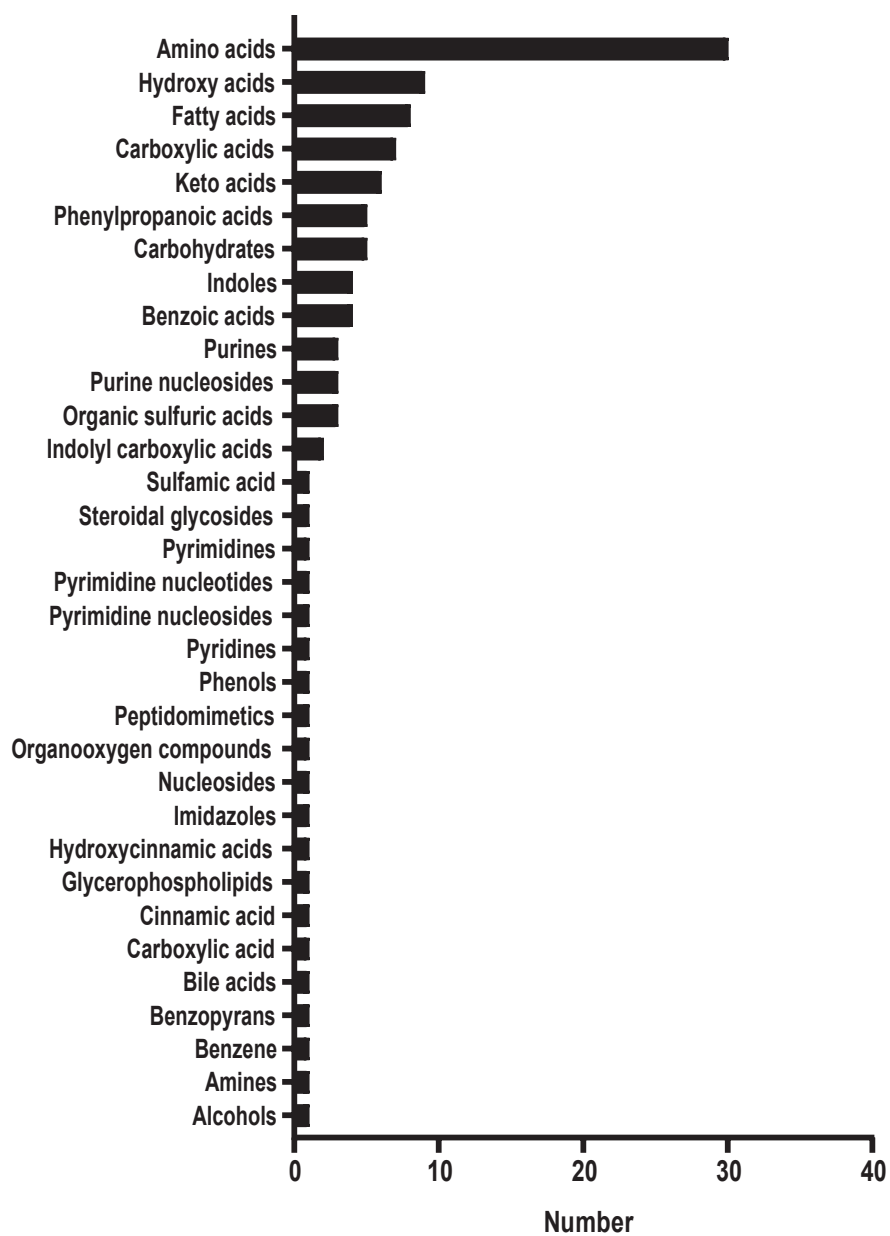**B**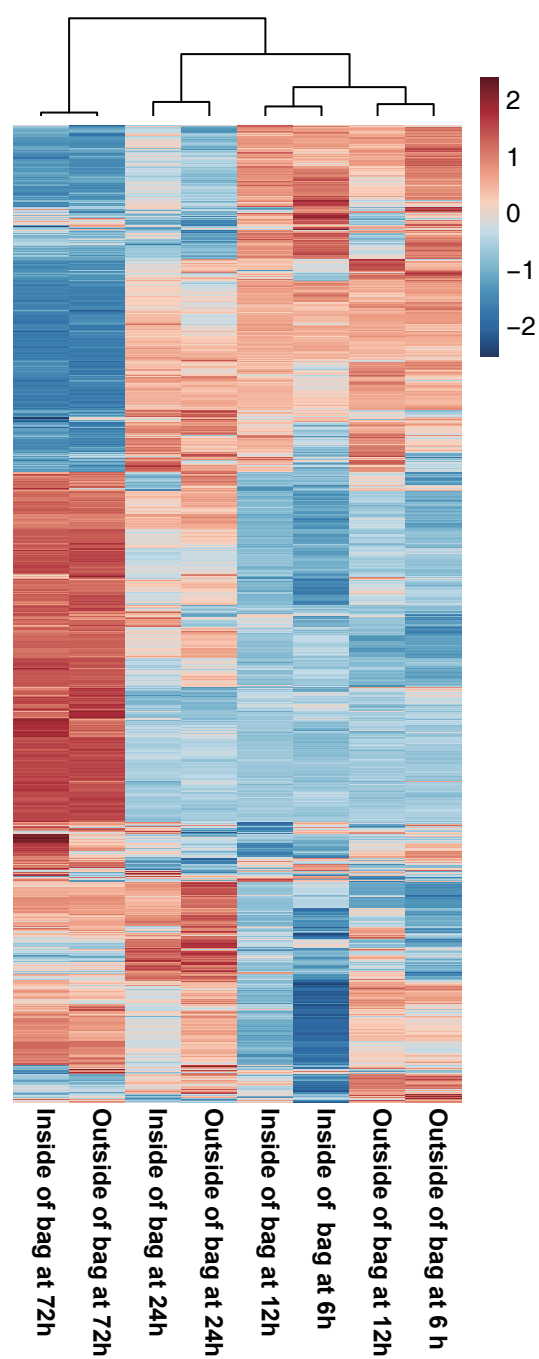**C**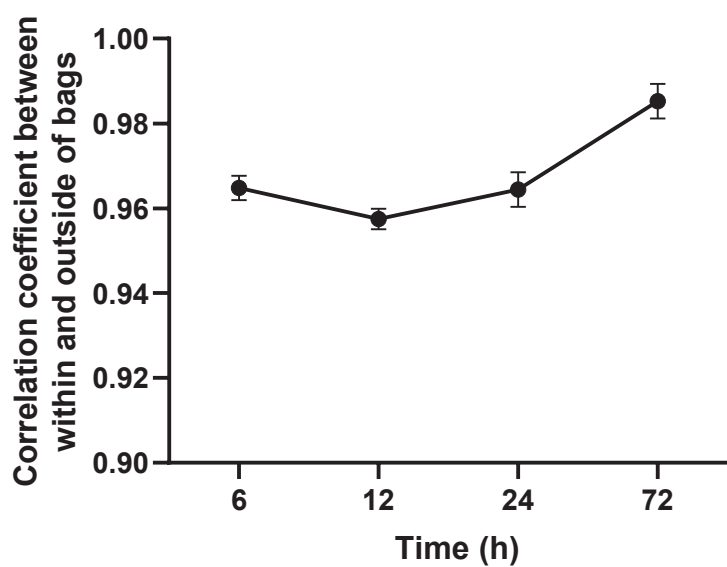

Supplement: Supplementary file 8 — Additional file 7: Supplementary Fig. 2. Microbial metabolites inside and outside of the dialysis bags. A. The identified metabolites inside the dialysis bags; B. A heatmap of the metabolite profiles at each incubation time both inside and outside of the dialysis bags; C. Correlation of metabolite profiles between insides and outside the dialysis bags. [file 40168_2023_1510_MOESM7_ESM.pdf]

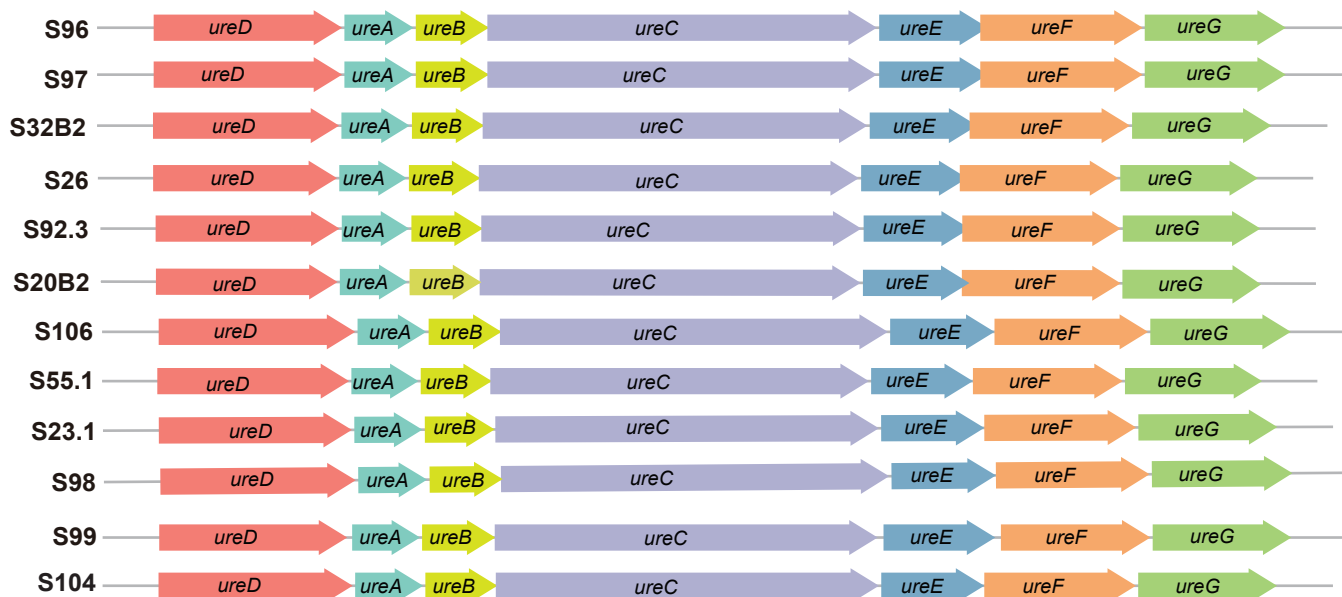

Type I

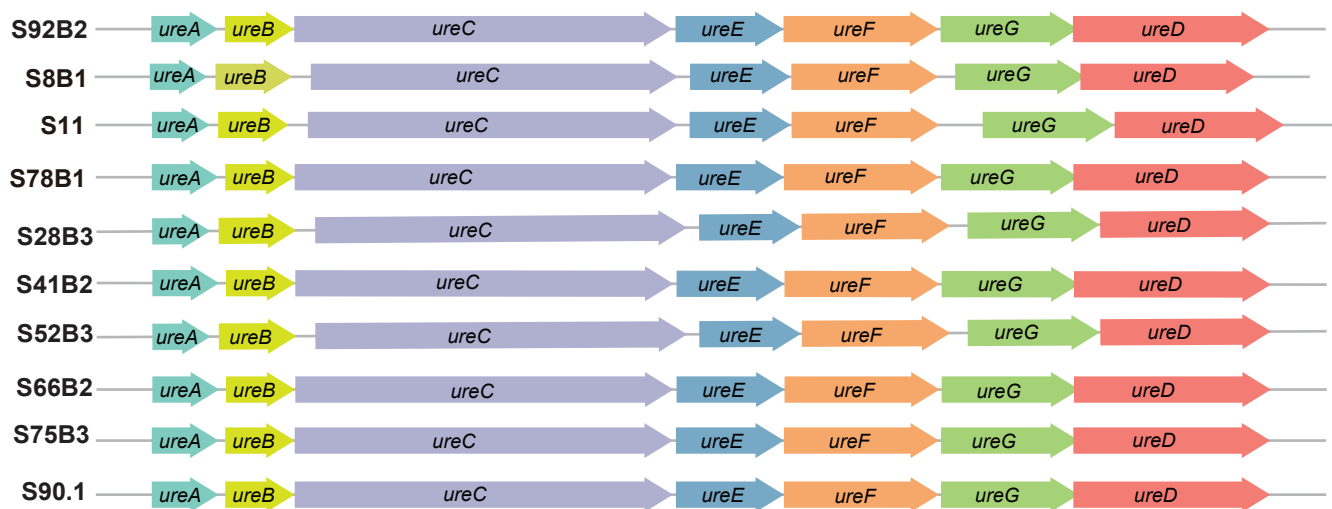

Type II

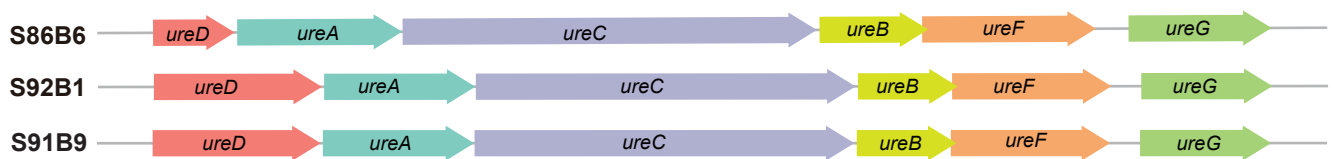

Type III

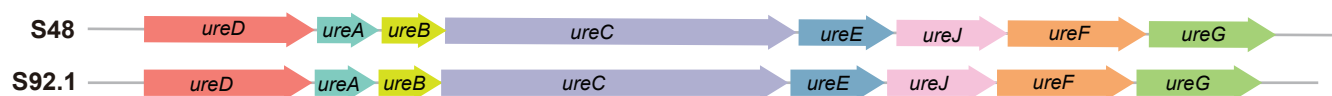

Type IV

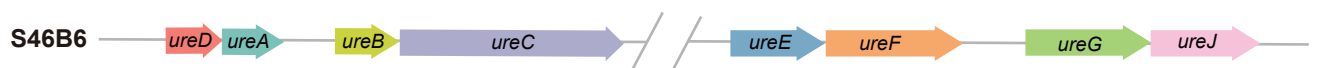

Type V

Supplement: Supplementary file 11 — Additional file 10: Supplementary Fig. 5. Urease gene clusters identified among the ureolytic isolates. [file 40168_2023_1510_MOESM10_ESM.pdf]

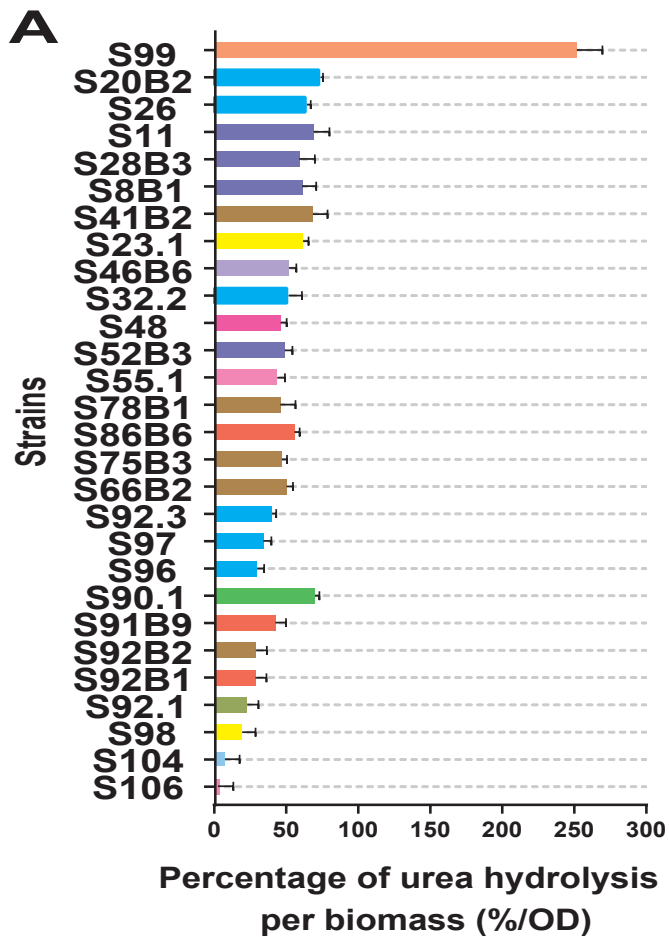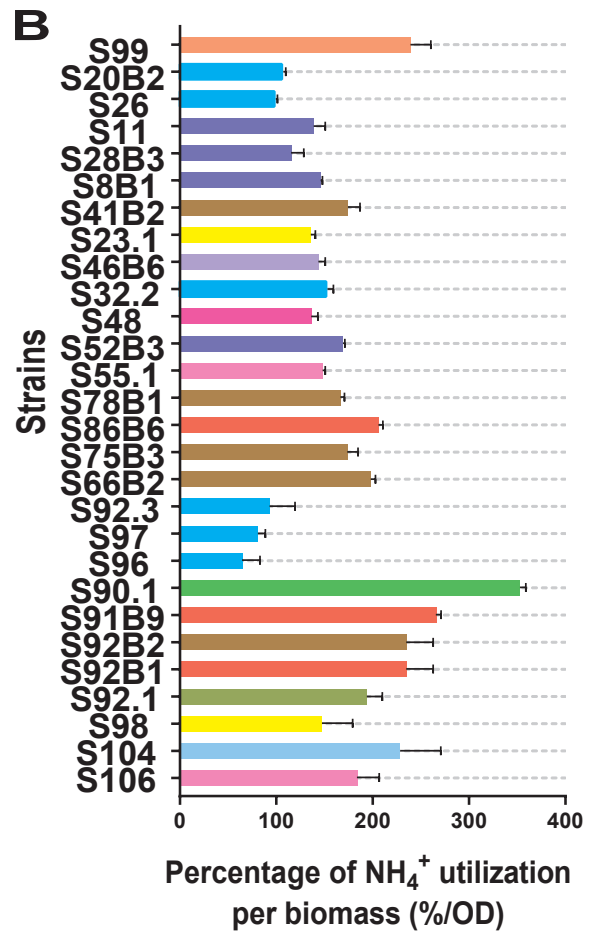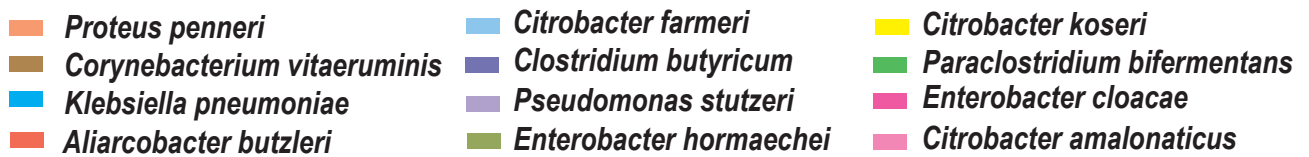

Supplement: Supplementary file 12 — Additional file 11: Supplementary Fig. 6. Urea hydrolysis rates (A) and urea-utilization rates of each ureolytic isolate per unit of culture optical density (OD, n = 3). [file 40168_2023_1510_MOESM11_ESM.pdf]

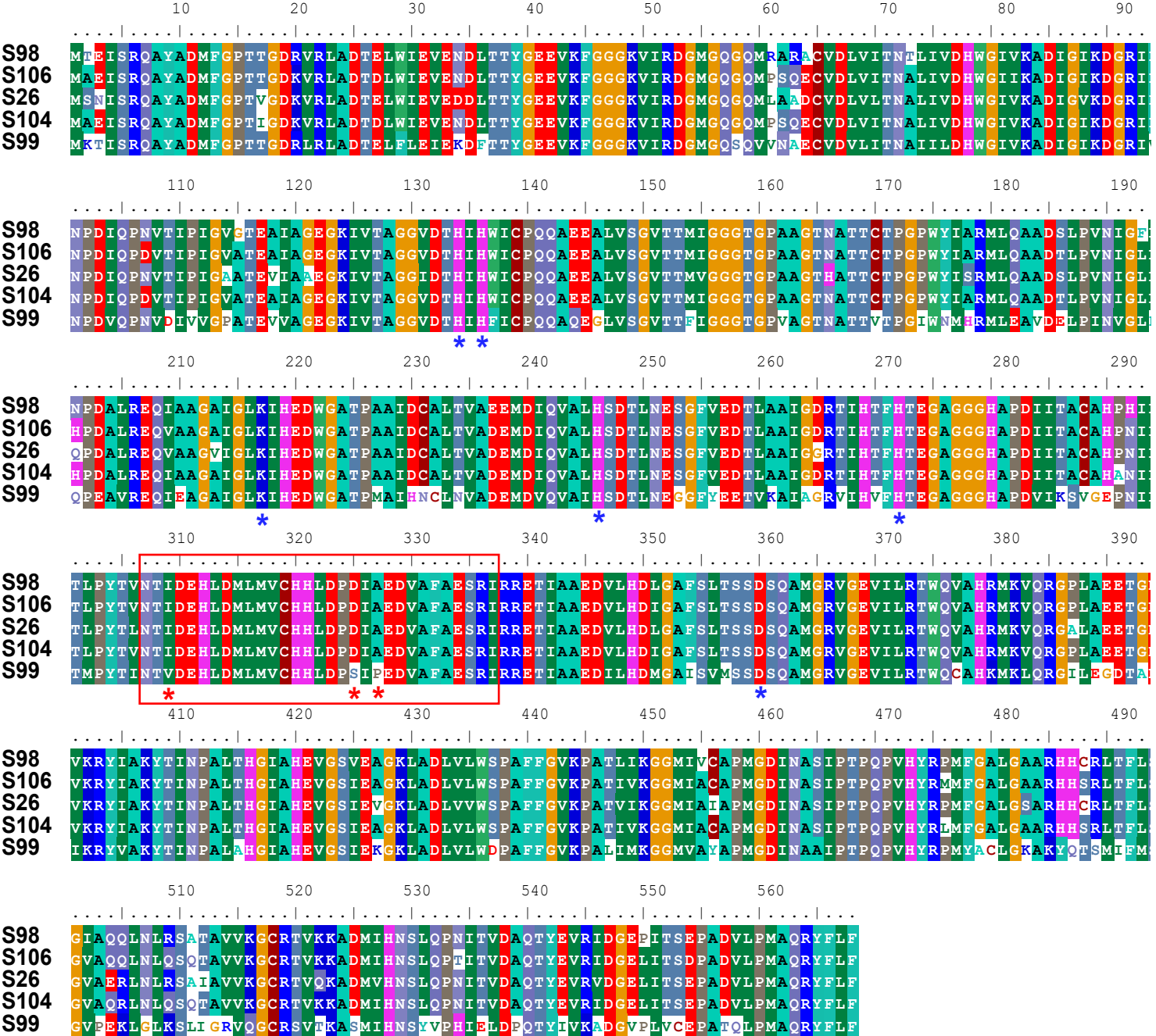

Supplement: Supplementary file 13 — Additional file 12: Supplementary Fig. 7. Sequence alignment of UreC of type I urease gene cluster. [file 40168_2023_1510_MOESM12_ESM.pdf]
