# Supplementary material for: Functional gene-guided enrichment plus in situ microsphere cultivation enables isolation of new crucial ureolytic bacteria from the rumen of cattle
Source: Microbiome. 2023 Apr 15;11:76. doi: 10.1186/s40168-023-01510-4 (PMC10105427; doi:10.1186/s40168-023-01510-4)
Supplement: Supplementary file 9 — Additional file 8: Supplementary Fig. 3. Circular diagrams of the core and unique gene clusters of the ureolytic isolates of individual species. [file 40168_2023_1510_MOESM8_ESM.pdf]

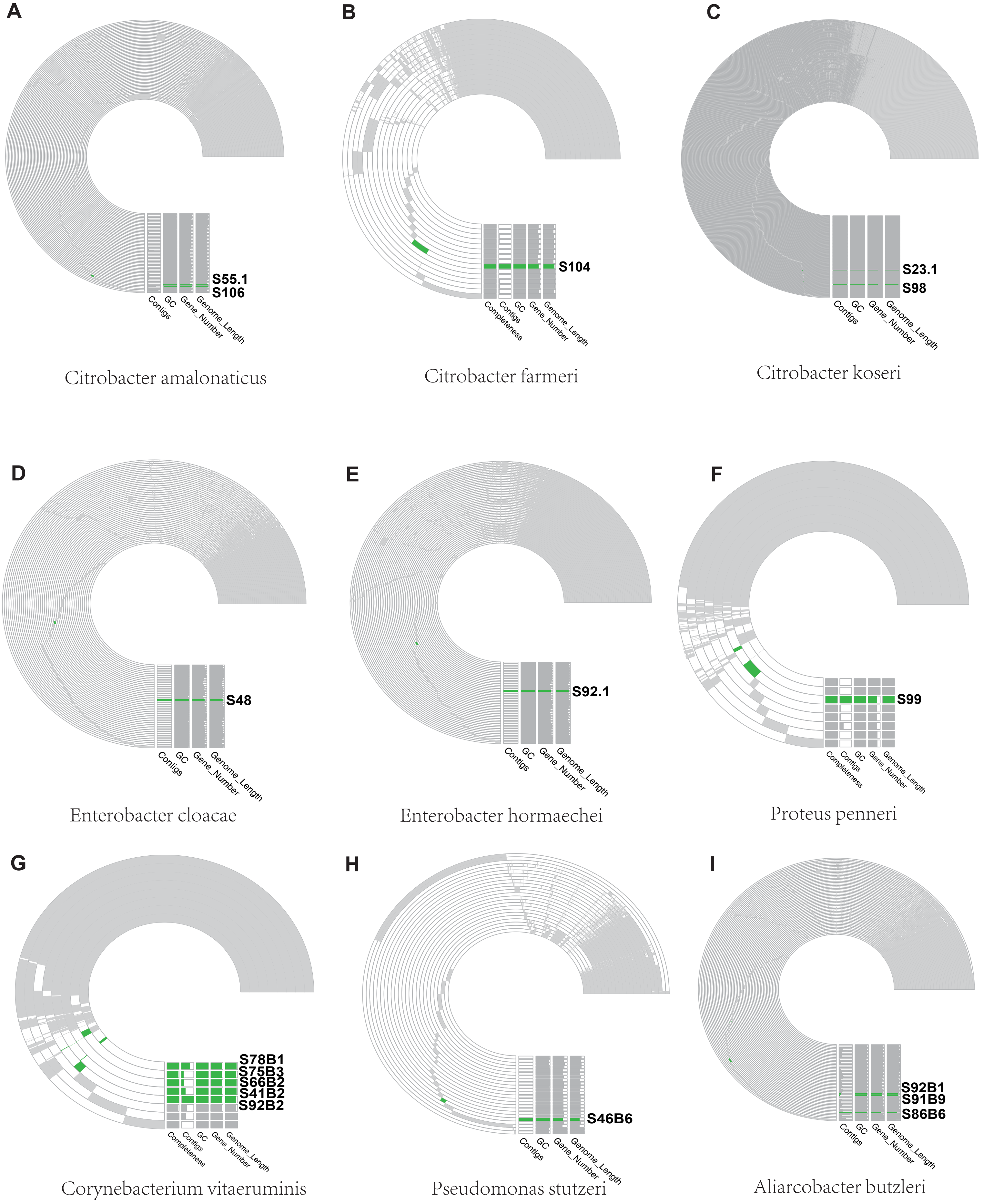
A
S55.1
S106
Contigs
GC
Gene_Number
Genome_Length
Citrobacter amalonaticus
B
S104
Completeness
Contigs
GC
Gene_Number
Genome_Length
Citrobacter farmeri
C
S23.1
S98
Contigs
GC
Gene_Number
Genome_Length
Citrobacter koseri
D
S48
Contigs
GC
Gene_Number
Genome_Length
Enterobacter cloacae
E
S92.1
Contigs
GC
Gene_Number
Genome_Length
Enterobacter hormaechei
F
S99
Completeness
Contigs
GC
Gene_Number
Genome_Length
Proteus penneri
G
S78B1
S75B3
S66B2
S41B2
S92B2
Completeness
Contigs
GC
Gene_Number
Genome_Length
Corynebacterium vitaeruminis
H
S46B6
Contigs
GC
Gene_Number
Genome_Length
Pseudomonas stutzeri
I
S92B1
S91B9
S86B6
Contigs
GC
Gene_Number
Genome_Length
Aliarcobacter butzleri
