# Supplementary material for: Functional gene-guided enrichment plus in situ microsphere cultivation enables isolation of new crucial ureolytic bacteria from the rumen of cattle
Source: Microbiome. 2023 Apr 15;11:76. doi: 10.1186/s40168-023-01510-4 (PMC10105427; doi:10.1186/s40168-023-01510-4)
Supplement: Supplementary file 10 — Additional file 9: Supplementary Fig. 4. Pan-genome profiles of ureolytic isolates. Pan-genome (blue) and core-genome (red) sizes were predicted based on all the strains of individual species. [file 40168_2023_1510_MOESM9_ESM.pdf]

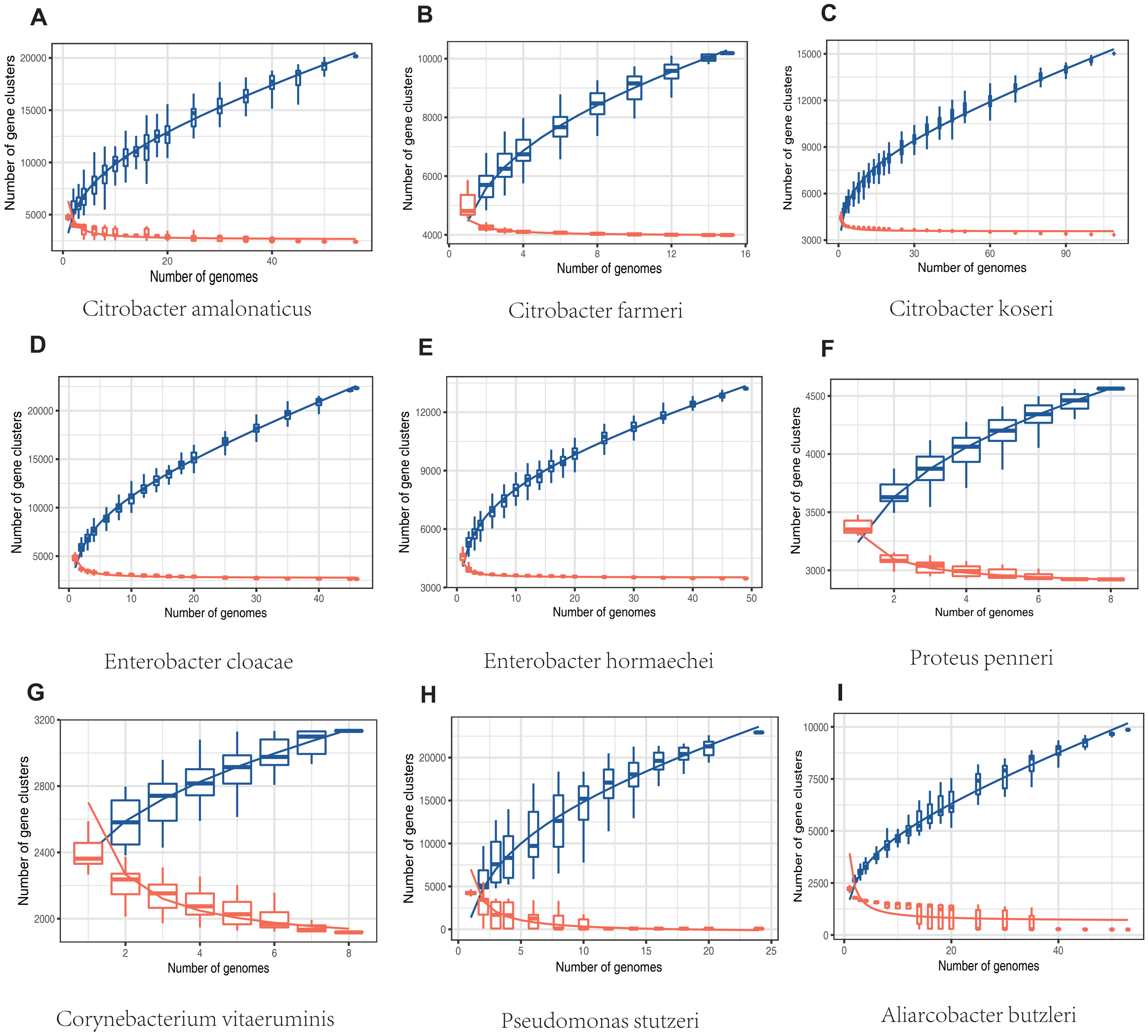
A
B
C
D
E
F
G
H
I
Number of gene clusters
Number of genomes
Citrobacter amalonaticus
Citrobacter farmeri
Citrobacter koseri
Enterobacter cloacae
Enterobacter hormaechei
Proteus penneri
Corynebacterium vitaeruminis
Pseudomonas stutzeri
Aliarcobacter butzleri
